# Comprehensive Analysis of Antioxidant Compounds from *Lippia citriodora* and *Hibiscus sabdariffa* Green Extracts Attained by Response Surface Methodology

**DOI:** 10.3390/antiox9121175

**Published:** 2020-11-25

**Authors:** María del Carmen Villegas-Aguilar, Francisco Javier Leyva-Jiménez, María de la Luz Cádiz-Gurrea, Antonio Segura-Carretero, David Arráez-Román

**Affiliations:** 1 Department of Analytical Chemistry, University of Granada, 18071 Granada, Spain; marivillegas@ugr.es (M.d.C.V.-A.); ansegura@ugr.es (A.S.-C.); 2 Research and Development of Functional Food Centre (CIDAF), 18016 Granada, Spain; jleyva@cidaf.es

**Keywords:** phenolic compounds, response surface methodology, *Lippia citriodora*, *Hibiscus sabdariffa*, antioxidant, Microwave-Assisted Extraction (MAE), Pressurized Fluid Extraction (PLE)

## Abstract

Phenolic compounds have shown to have a high bioactive potential against various pathologies, postulating as an interesting alternative to manage some diseases. In this sense, both *Lippia citriodora* and *Hibiscus sabdariffa* are two botanical sources with a demonstrated high bioactive potential, in which their antioxidant capacity stands out. In this work, the optimization of the extraction conditions for the recovery of phytochemicals from *L. citriodora* leaves and *H. sabdariffa* calyces has been carried out using Response Surface Methodologies (RSM) considering their total polar compounds measured by HPLC-ESI-TOF/MS and Folin-Ciocalteu assay, and its antioxidant capacity evaluated by Ferric Reducing Antioxidant Power (FRAP) and Trolox Equivalent Antioxidant Capacity (TEAC) assays. The results showed that to maximize the antioxidant capacity in *H. sabdariffa*, a moderate temperature and high ethanol percentage are needed, while a low temperature and a high percentage of ethanol are needed in *L. citriodora*. In addition, with the results obtained in the multiple response analysis, it is possible to affirm the importance of this type of analysis to develop functional ingredients, taking into account both total content of phenolic compounds and their bioactivity. Furthermore, as confirmed in this study, these analyses can be extrapolated in different techniques and in different matrices, with phenolic compounds from different families being important to develop new high added value products for food, pharmaceutical or cosmetic industries.

## 1. Introduction

In recent years, both nutraceuticals and functional food have received increasing attention from the scientific community, consumers and food manufacturers [1]. This growing interest is related to their composition and health benefits. These benefits are attributed by the source used during their manufacturing, which are, generally, botanicals. In fact, there is a huge variety of botanicals that have been reported to exhibit biological properties such as antioxidant or anti-inflammatory [2,3,4]. In this sense, plants such as *Hibiscus sabdariffa* and *Lippia citriodora* stand out among an extensive list, since both have been traditionally used to prepare herbal drinks with numerous health benefits. Several of these beneficial properties have been related to the bioactive compounds present in their composition, mainly, phenolic compounds [5,6].

Several reports of *H. sabdariffa* have demonstrated its potential effects such as antioxidant [6], anti-obesity [7], hypotensive [8], antidiabetic [7], hypocholesterolemic [9], immunomodulatory [10], hepatoprotective [11] and diuretic [10]. In the case of *L. citriodora*, its antioxidant [10], anti-inflammatory [12], antimicrobial [13] and anticancer [10] properties, among others, have been also highlighted. These biological properties are especially related to the phenolic compounds, which are secondary metabolites contained in their leaves, stems or flowers.

In spite of having great benefits on the human health, the amount of these compounds are usually reduced, being necessary an adequate extraction technique to solve several issues: (i) to avoid or decrease their degradation during the extraction processes and (ii) to concentrate them or to perform selective extractions. There are numerous extraction techniques to attain enriched extracts in phenolic compounds from botanical sources, but currently the non-conventional or advanced techniques stand out, due to the fact that they have fewer extraction times, lesser consumption of solvents (which may be Green and Generally Recognized As Safe, GRAS) and energy. For these reasons, they have been recognized as environmentally friendly techniques, being more efficient than conventional ones [14,15]. Some examples of advanced extraction techniques are Microwave-Assisted Extraction (MAE) and Pressurized Fluid Extraction (PLE).

Regardless of the extraction technique selected, during an extraction process, the adjustment of the extraction parameters is an important step since, depending on the targets desired, the best extraction conditions may have a determinant influence. In the last decade, the use of Response Surface Methodologies (RSM) has been applied with the purpose of optimizing the extraction conditions considering the aims desired, enhancing the efficiency of advanced extraction technologies [14,16,17].

There is a large number of studies that propose different extraction methods to obtain enriched extracts in specific bioactive compounds by RSM and its possible incorporation in functional food or in nutraceuticals [14,17,18,19]. However, the health benefits of the extracts lie in their bioactivity, which is influenced by synergies and antagonism between compounds. Due to this, innovative researches are focused not only on optimizing the extraction and composition performance; rather, they use variables that measure bioactivity [20,21].

The aim of this study was to optimize the extraction conditions for the recovery of phytochemicals from *L. citriodora* leaves and *H. sabdariffa* calyces, considering their total polar content and antioxidant capacity, by different methods in order to obtain ingredients with high antioxidant potential. In addition, the optimization of extraction processes by MAE and PLE was compared, considering, on one hand, the total content of polar compounds measured by high-performance liquid chromatography coupled to mass spectrometry (HPLC-ESI-TOF/MS) and, on the other hand, the bioactivity measured by different in vitro tests.

The optimization was carried out using an RSM based on Central Composite Design 2^3^ (CCD) model with 16 experiments including center and star points. The independent variables were temperature, extraction time and percentage of solvent (ethanol and water) and the response variables were total phenolic compounds measured by HPLC-ESI-TOF/MS and by Folin-Ciocalteu assay, antioxidant capacity evaluated by Ferric Reducing Antioxidant Power (FRAP) assay and Trolox Equivalent Antioxidant Capacity (TEAC).

## 2. Materials and Methods

### 2.1. Chemicals and Reagents

For extractions and solutions, ultrapure water was obtained with a Milli-Q system Millipore (Bedford, MA, USA) and absolute ethanol was purchased from VWR chemicals (Radnor, PA, USA). To measure the antioxidant capacity and total phenolic content, the following reagents were provided from the indicated suppliers: ABTS (2,2′-azinobis (3-ethylbenzothiazoline-6-sulphonate)), ferric sulfate, Folin–Ciocalteu reagent, potassium persulfate, TPTZ (2,4,6-Tris(2-pyridyl)-s-triazine), Trolox (6-hydroxy-2,5,7,8-tetramethylchroman-2-carboxylic acid) from Sigma-Aldrich (St. Louis, MO, USA). Acetic acid and methanol were purchased from Fluka (Sigma-Aldrich, Steinheim, Germany) and Lab-Scan (Gliwice, Poland), respectively, whereas gallic acid, sodium acetate, ferric chloride, hydrochloric acid, trihydrated sodium acetate and sodium carbonate were obtained from Panreac (Barcelona, Spain).

The analytical procedures were performed using LC-MS grade methanol and acetic acid, which were purchased from Fisher Chemicals (Waltham, MA, USA) and Sigma-Aldrich (Steinheim, Germany), respectively.

### 2.2. Sample Preparation

*H. sabdariffa* calyces and *L. citriodora* leaves were provided by Monteloeder (Alicante, Spain) whose calyces and leaves were milled using an ultra-centrifugal mill ZM 200 (Retsch GmbH, Haan, Germany). The resulting powder was stored in darkness and kept at room temperature until extraction. 

### 2.3. Extraction of Phenolic Compounds and Other Polar Compounds from H. sabdariffa Calyces

MAE was carried out in according to the methodology used by Pimentel-Moral et al. [17] in a modular microwave extraction system Multiwave 3000 SOLV (Anton Paar GmbH, Graz, Austria). Briefly, 3 g of *H. sabdariffa* dried calyces were added into the vessels together with 30 mL of water-ethanol mixtures. After cooling, samples were centrifuged at 17,000× *g* for 15 min in a centrifuge (Sorvall ST 16 R, Thermo Scientific, Leicestershire, UK) and the supernatants were dried under vacuum in a Savant^TM^ SpeedVac Concentrator SC250 EXP (Thermo analysis Scientific, Sunnyvale, CA, USA). The extracts were stored at −20 °C until further use. Prior to use, the dry extracts were reconstituted in the same extraction solvent mixture to a concentration of 10 mg/mL and filtered with single-use syringe filters (0.20 µm pore size).

### 2.4. Extraction of Phenolic Compounds and Other Polar Compounds from L. citriodra Leaves

PLE was run according to the methodology used by Leyva-Jiménez et al. [14] with a pressurized liquid extractor (ASE™ 350 system, Dionex, Sunnyvale, CA, USA). Briefly, for each extraction, 5 g of sample were mixed with 10 g of sea sand and loaded onto 33 mL stainless-steel extraction cells. After extraction, the extracts obtained were ice-cold to achieve a temperature of 20–25 °C and centrifuged at 17,000× *g* for 15 min in a centrifuge (Sorvall ST 16 R, Thermo Scientific, Leicestershire, UK). The supernatants were dried under vacuum in a Savant^TM^ SpeedVac Concentrator SC250 EXP (Thermo analysis Scientific, Sunnyvale, CA, USA) and they were stored at −20 °C until further use. Prior to use, the dry extracts were reconstituted with the same extraction solvent mixture to a concentration of 10 mg/mL and filtered with single-use syringe filters (0.20 µm pore size).

### 2.5. Experimental Design

For both MAE and PLE extractions, the experimental conditions were defined according to an RSM using a CCD 2^3^ model with axial and center points. RSM was designed with Statgraphics Centurion XVI software (Statpoint Technologies (Warrenton, VA, USA). In the MAE of *H. sabdariffa* calyces, the independent variables were temperature (50, 100, 150 °C), extraction time (5, 12.5, 20 min) and percentage of ethanol in the hydro-alcoholic mixture (15%, 45%, 75%). Conditions were selected based on Pimentel-Moral et al. [17]. A total of 16 experimental conditions were obtained (Table S1) that were carried out in a randomized order.

In the PLE of *L. citriodora* leaves, the independent variables were temperature (40, 110, 180 °C), extraction time (5, 12.5, 20 min) and percentage of ethanol in the hydro-alcoholic (15%, 50%, 85%). Conditions were selected based on Leyva-Jiménez et al. [14]. A total of 16 experimental conditions were obtained (Table S2) that were carried out in a randomized order.

Total polar compounds were quantified by HPLC-ESI-TOF/MS according to Pimentel-Moral et al. in *H. sabdariffa* extracts and adapted from Leyva-Jiménez et al. in the case of *L. citriodora* extracts; total phenolic compounds were measured by Folin-Ciocalteu assay, whereas antioxidant capacity was evaluated using FRAP and by TEAC. The results of each assay were chosen as response variables in both models. The following second-order polynomial model was used to fit the experimental data (Equation (1)):

(1) $$Y=\;\beta_{0}+\;\overset{k}{\underset{\;i\;=\;1}{\sum }}\beta_{i}X_{i}+\;\overset{k}{\underset{i=1\;}{\sum }}\beta_{ii}\;X_{i}^{2}+\;\overset{k}{\underset{i=1}{\sum }}\overset{k}{\underset{j=i+1}{\sum }}\beta_{ij}\;X_{i}\;X_{j}$$

*Y* represents the predicted response; *β*_0_ is a constant coefficient that fixes the response at the central point of the experiments, and *β_i_*, *β_ii_* and *β_ij_* are the regression coefficients of the linear, quadratic and interaction terms, respectively; *X_i_* and *X_j_* represent the value of the independent variables. The parameters were considered to evaluate the model adequacy: regression coefficient (*R*^2^), coefficient of variation (CV) and model value.

### 2.6. Total Phenolic Content by Folin Ciocalteu

The total phenolic content (TPC) was measured by Folin-Ciocalteu method with some modifications [22]. *L. citriodora* and *H. sabdariffa* extracts were dissolved in ethanol–water (1:1) (different concentrations were tested). The absorbance measurement was carried out on a Synergy Mx Monochromator-Based Multi-Mode Micro plate reader (Bio-Tek Instruments Inc., Winooski, VT, USA) using 96-well polystyrene microplates. Phenol content was calculated based on the calibration curves of Gallic Acid and expressed as mg Gallic acid equivalents/g of dry extract. The experiments were made in triplicate.

### 2.7. Antioxidant Activity Assays

The antioxidant activity of *L. citriodora* and *H. sabdariffa* extracts were evaluate using two methods based on single-electron transfer (SET): FRAP and TEAC assays. In both, fluorescence was measured with the same equipment mentioned above.

#### 2.7.1. Ferric Reducing Antioxidant Power

The FRAP assay was carried out following the method described by Benzie and Strain [23]. FRAP values were calculated measuring the absorbance at 593 nm in a microplate reader. A standard curve of FeSO_4_·7H_2_O was assessed and results were expressed as mmol FeSO_4_ equivalents/g of dry extract. The experiments were made in triplicate.

#### 2.7.2. Trolox Equivalent Antioxidant Capacity

The TEAC assay was originally described by Miller et al. and was performed with some modifications according to Cádiz-Gurrea et al. [24,25]. TEAC values were calculated using Trolox as standard and reading absorbance at 734 nm in a microplate reader. The results were expressed in mmol Trolox equivalents/g of dry extract. The experiments were made in triplicate.

### 2.8. HPLC-MS Analysis

The qualitative characterization of *H. sabdariffa* and *L. citriodora* extracts was carried out using a RRPC 1200 series (Agilent Technologies, Palo Alto, CA, USA) following the methods reported by Pimentel-Moral et al. [17] and Leyva-Jiménez et al. [14], respectively, in order to ensure the reproducibility.

## 3. Results and Discussion

### 3.1. Total Polar Compounds, Total Phenolic Contents and Antioxidant Activities of H. sabdariffa by Microwave-Assisted Extraction (MAE)

The analysis of the beneficial properties of plant extracts can be approached from different points of view. For instance, attention can be focused on the composition of bioactive compounds as evaluating a wide group of compounds as polar compounds or more specific as phenolic compounds, since these are responsible for the vast majority of beneficial properties reported. On the other hand, the studies can be focus on the bioactivity, such as antioxidant capacity of the extracts that may be mostly associated with the phenolic profile of this type of polar extractions, but taking into account the positive and negative synergies that have influence in the antioxidant properties. It is necessary to consider that the results of each applied assay to evaluate this bioactivity may change according to the chosen technique. For these reasons, this work pretends to approach the relationship between phytochemicals contained in the extracts and their antioxidant activity in order to establish optimal conditions for the obtainment of functional ingredients.

In this sense, Table 1 shows the results of the total content of polar compounds adapted from Pimentel-Moral et al. [17], the TPC measured by the Folin-Ciocalteu and the antioxidant activity obtained by the FRAP and TEAC assays for each of the experimental conditions of MAE extraction of *H. sabdariffa* calyces. In this case, the extraction yield has been used to ensure the reproducibility of the extractions with those made by Pimentel-Moral et al. [17], noting that there were no non-significant differences (data not shown). Table 2 shows the major compounds tentatively identified in *H. sabdariffa* calyces by HPLC-ESI-TOF-MS following the method reported by Pimentel-Moral et al. [17].

As can be seen in Table 1, there is no condition in which all the response variables reach the maximum; however, when attention is paid to the condition with the lowest value for the response variables, it is observed that condition 10 has the lowest value for the four response variables. These results indicate that the composition of the *H. sabdariffa* extracts have an important influence on the antioxidant activity.

The results of Total Polar Compounds variable ranged from 201.466 ± 5.356 mg of total polar compounds/g of dry extract (run 11) to 50.027 ± 1.107 mg total polar compounds/g of dry extract (run 10); the extraction conditions under these conditions were 164 °C, 12.5 min and 45% ethanol, and 150 °C, 20 min and 15% ethanol, respectively. For Folin-Ciocalteu response, the condition with the highest value was run 8 with 78.679 ± 2.591 mg Gallic acid equivalents/g of dry extract under the conditions 150 °C, 5 min, 75% ethanol; for FRAP, conditions 5 and 15 with 0.995 ± 0.034 and 0.995 ± 0.018 mmol FeSO_4_ equivalents/g of dry extract under 100 °C, and 45% ethanol, and for TEAC, condition 11 gave 0.285 ± 0.010 mmol Trolox equivalents/g of dry extract. The results achieved by condition 11 offered higher values for total polar compounds as well as TEAC assay, which may highlight the synergic effect of polar compounds in the antioxidant activity. Some examples of this synergistic effect on antioxidant activity between phenolic compounds was evaluated by Skroza et al., in which they observed that in different tests to measure antioxidant capacity there was a synergistic effect between resveratrol and other phenols such as catechin and caffeic acid [26]. Also in this sense, in the case of the TEAC assay, Sanchez-Marzo et al. tested different fractions of compounds present in *L. citriodora*, checking the fraction with a combination of Apigenin-7-diglucuronide, Verbascoside, Isoverbascoside and Forsythoside A presented higher values in both FRAP and TEAC, compared to other fractions with different combinations or with isolated compounds. Thus, this demonstrates the synergistic effect of the compounds present in this fraction [27]. As mentioned above, condition 10 gave the lowest values for all responses evaluated.

In spite of run 11 revealing the highest total polar compounds in the evaluated design, the value reached in Folin-Ciocalteu by condition 8 was the highest value. This unexpected value may be due to the fact that Folin-Ciocalteu reagent not only reacts with phenolic compounds, which are included within polar compounds, but also reacts with others such as proteins, thiols, many vitamin derivatives and inorganic ions Fe^2+^, Mn^2+^, I^−^ and SO_3_^2−^ [28]. According to these results, higher percentage of ethanol from 45% (run 11) to 75% (run 8) allowed greater extraction of pigments such as chlorophyll, carotenoids and xanthophylls [29], which can be conjugated with proteins and have some of the aforementioned ions in their structure, reacting with the Folin-Ciocalteu reagent. Additionally, the lowest and least maintained temperature over time of condition 8 compared to 11 prevents the degradation of certain proteins and vitamins, explaining the high value for Folin-Ciocalteu of condition 8 [30].

Considering the response variables to evaluate the antioxidant capacity, condition 11 showed the highest TEAC value and the greatest total polar compounds. Moreover, the values of organic acids, phenolic acids, flavonoids and anthocyanins showed the highest, so that in relation to TEAC the greatest concentration of compounds is directly related to the highest antioxidant activity. However, in the case of FRAP, the results differ, since there were two conditions that revealed greater values than conditions 11, 5 and 15 that were performed with 45% of ethanol. Moreover, the temperature had an important relevance since, while in condition 11 the extraction temperature was 164 °C, in 5 and 15 it was 100 °C. These results may be occasioned by the thermal degradation of compounds like flavonoids. Quercetin and its derivatives are flavonoids that were found in *H. sabdariffa* extracts, and their abilities to reduce iron in the FRAP test compared to other flavonoids have been reported [31]. According to Pimentel-Moral et al., in conditions 5 and 15, the content of quercetin-3-rutinoside was greater than condition 11, which could explain the higher value in FRAP of these two conditions [17].

### 3.2. Total Polar Compounds, Total Phenolic Contents and In Vitro Antioxidant Activities of L. citriodora Pressurized Fluid Extraction (PLE)

Table 3 shows the results attained after evaluating the PLE *L. citriodora* extracts, where the results of total content of polar compounds were adapted from Leyva-Jiménez et al. [14], whereas the TPC was measured applying the Folin-Ciocalteu assay and the antioxidant activity performing FRAP and TEAC assays. In the same way that was mentioned previously for *H. sabdariffa*, the extraction yield has been the parameter used to compare the extractions carried out in this study with those made by Leyva-Jiménez et al. [14], noting that there were no non-significant differences (data not shown). Table 4 shows the major compounds tentatively identified in *L. citriodora* leaves by HPLC-ESI-TOF-MS following the method described by Leyva-Jiménez et al. [14].

Concerning the FRAP response variable, the condition with the highest value coincided with Total Polar Compounds and Folin-Ciocalteu, pointing out a positive relationship between the amount of phenolic compounds and the ability to reduce the iron ion through the transfer of electrons. Furthermore, in accordance with Leyva-Jiménez et al., run 2 showed the highest amount of verbascoside, which may explain the antioxidant results of this run. This could be corroborated by various studies that show the positive relationship between the amount of verbascoside in the matrix and the highest capacity in the FRAP test [27,32].

Nevertheless, for the TEAC response variable, the condition run 14 exhibited the highest value. These results may be explained due to synergistic relationships between the phytochemicals contained in this extract and showing that the amounts of polar/phenolic compounds were not related with reduction of the ABTS cation radical. There are studies that show that there is not always a direct relationship between the relative concentration of a compound or certain compounds with the bioactivity, but in many situations, synergistic or antagonist relationships are determinant to the bioactive potential of the extracts obtained from plants [33].

Lastly, it is necessary to note that the minimum value of all responses evaluated was achieved after applying higher temperatures (above 180 °C). This may be due to the fact that there were compounds that are thermolabile at high temperatures and therefore could be degraded [12].

### 3.3. RSM Analysis of H. sabdariffa Calyces MAE and L. citriodora Leaves PLE Conditions

#### 3.3.1. Model Fitting Parameters

To maximize the response variables for each model, an analysis of variance (ANOVA) was carried out for each response to discern the adjustment of the results and optimize the statistical model. To achieve this goal, several fitting parameters were used, which were evaluated to determine the adequacy of the model. The results were fitted to a quadratic polynomial model (Equations (S1) and (S2)) and regression coefficients were generated using the least squares method (MLS). The first parameter to evaluate the adequacy of the model was the regression coefficient (R^2^), in which values above 70% are considered acceptable [34]. This value explains the variability of the data which may be explained with the proposed model. The coefficient of variation (CV) was also used, a lower value (<10%) indicates a good reproducibility of the investigated systems and a value between 11% and 20%, indicates an acceptable variation; hence, CV shows the dispersion of data and a small value indicated high reproducibility between the predicted and experimental values [35]. Finally, the adequacy of the model (model value) was also used as approach to discern the good choice of the design. Table 5 shows the parameters mentioned to validate the model in *H. sabdariffa* and Table 6 the results of the ANOVA for this matrix. Table 7 shows the parameters mentioned to validate the model in *L. citriodora* and Table 8 the results of the ANOVA for this matrix.

#### 3.3.2. *H. sabdariffa* Calyces MAE Optimization

Regarding the evaluated fitting parameters (Table 5), Folin-Ciocalteu, FRAP and TEAC response variables showed R^2^ values greater than 70% (93.7%, 78.4% and 74.0%, respectively), hence revealing a good explanation of the obtained results. Moreover, the adequacy of the model of each response gave significant values (*p* ≤ 0.05) demonstrating a good adequacy, and the CV was below 20 and close to 10, revealing an acceptable reproducibility of the results. Nevertheless, Total Polar Compound did not present a good approximation to the experimental conditions since neither the result of *R*^2^ model value nor CV was acceptable.

Considering the effects of the independent variables, it is noteworthy that the % ethanol had a significant influence for the Folin-Ciocalteu, FRAP and TEAC response. Furthermore, the optimization of these responses (Table 6) was achieved when the ethanol used was between 67% and 83%. This could be explained because water, despite having higher dielectric constant, has a dissipation factor significantly lower than ethanol. In this sense, a high dissipation factor improves the passage of microwave energy through the solvent and the dissipation of microwave energy into heat, as is the case with ethanol [36]. Despite this characteristic, its ability to absorb energy is low. This causes less heat transfer from the solvent to the sample [37], requiring water in the solvent mixture to improve this situation. With these observations, a higher extraction yield of phenolic compounds were obtaining using 80% of ethanol in the mixture, comparing with water and other mixtures of ethanol-water and methanol-water, which coincide with the results obtained in the present study [36].

Furthermore, Total Polar Compounds and Folin-Ciocalteu showed the optimal temperatures at 164 °C and the extraction time was 13 and 3 min, respectively. Nevertheless, the optimal temperature for FRAP was 62 °C, while for TEAC it was 118 °C, and the optimal extraction time was 12 and 13 min, respectively. In these cases, the temperature necessary to obtain the optimal values of FRAP and TEAC enabled the extraction of thermo sensitive compounds as flavonoids. These flavonoids had a greater affinity for reducing iron in FRAP compared to the TEAC test based on the ABTS radical. These flavonoids had a higher affinity for reducing iron in FRAP compared to the ABTS radical-based TEAC test. This result may be explained considering the results attained by Bahorun et al. and Csepregi et al., which conducted FRAP and TEAC tests for different types of flavonoids. The results of their studied revealed that particularly quercetin and quercetin-3-*O*-rutinoside, very abundant in *H. sabdariffa*, had higher values for FRAP than for TEAC [38,39]. This can be explained since the presence an aliphatic substituent and a double bond on a catechol ring is positively associated with the iron ion reduction [40].

#### 3.3.3. *L. citriodora* Leaves PLE Model

Table 7 shows variable responses for *L. citriodora* PLE extractions, which gave R^2^ values above 70%, reaching values near 100% in Total Polar Compounds and Folin-Ciocalteu responses: 95.5% and 91.8%, respectively. With regard to the adequacy of the model, it can be observed that for Total Polar Compounds and Folin-Ciocalteu it was significant (*p* ≤ 0.05), while for the variables FRAP and TEAC, it was marginally significant (*p* ≤ 0.1). Finally, when the third parameter was analyzed, the CV was observed to be below 20% in the four variables. After analyzing these parameters, it can be considered that this model provided a good approximation and explanation of the behavior of each variable evaluated.

Regarding the effect of the independent variables, it is noted that for all response variables evaluated, temperature had a significant effect (*p* ≤ 0.05), except for TEAC, which made it marginally significant (*p* ≤ 0.1). The negative influence of temperature is exposed in Table 8, which confirms that the temperature necessary to optimize these variables was very low (from 20 to 25 °C), with the exception of the FRAP, which was moderate (71 °C). Ethanol percentage had a significant effect in Total Polar Compounds and Folin-Ciocalteu response variables, whereas for FRAP it was marginally significant. Table 8 demonstrates the positive significant effect exerted since for these three variables the optimal percentage was 95% ethanol. When the temperature and ethanol data are viewed together, it can be thought that the optimal low temperatures for extraction are combined with high percentages of ethanol to increase the solubility of the compounds. Both parameters, lower temperatures and higher concentrations of ethanol, may induce a better recovery of phytochemicals according to their polarity. Therefore, the compounds extracted under these conditions allowed greater reduction of iron ion in FRAP assay.

Conversely, TEAC presented an optimal percentage of ethanol that was very low (5%) (Table 8). This may be because the catechol group present in some phenolic compounds has a higher solubility in water [41]. As mentioned above, the catechol group is of great importance in the FRAP assay, but also in the TEAC assay. This catechol group has shown to have a fundamental role in the antioxidant capacity measured by TEAC, so phenolic compounds with more than one catechol group such as verbascoside, isoverbascoside and forsythoside A, which are very abundant in *L. citriodora*, will play a key role in the antioxidant capacity measured by TEAC [27]. Furthermore, another group present in *L. citriodora*, which are extracted in greater quantities with low percentages of ethanol [14], are the glucuronized flavonoids, in which the number of catechol groups is greater.

### 3.4. Multiple Response Optimization Analysis of H. sabdariffa Calyces MAE and L. citriodora Leaves PLE Conditions

After analyzing the fitting parameters and the effect of each factor in the responses evaluated, the optimization of extraction processes to maximize response variables, phenolic content and bioactivity, was investigated. The antioxidant activity responses revealed different optimal condition depending on the assay to evaluate this bioactivity. For this reason, it was carried out an evaluation of a multiple response analysis with the aim of maximizing both dependent variables, phenolic content and antioxidant capacity. For this analysis, the desirability function was applied. According to Yolmeh and Jafari, the desirability function was based on the idea that the “quality” of a product or process which has multiple quality characteristics; in other words, the desirability function explains the best conditions to achieve the optimum values of evaluated responses [42]. The method finds conditions that provide the “most desirable” response values.

Table 9 shows the Multiple Response Optimization for the extraction of *H. sabdariffa* calyces by MAE and for the extraction of *L. citriodora* leaves by PLE. In the case of *H. sabdariffa*, the response variable Total Polar Compounds was eliminated and was not considered for this analysis because, as mentioned in previous sections, the adjustment parameters for that response were not adequate. For both matrices, the evaluated model explains the variability of the data for the three response variables evaluated in the case of *H. sabdariffa*, and for the four response variables in *L. citriodora*, since in both analyses the optimal desirability parameter is about 1.

For *H. sabdariffa* with the focus on the Multiple Response Optimization to maximize Folin-Ciocalteu, FRAP and TEAC, the proposed optimal conditions were: temperature of 122.25 °C, % ethanol of 78.58% and a time of around 3 min. Considering these conditions, the optimal temperature was moderate and the extraction time presented an optimal short time. This can be explained due to the high content of flavonoids present in this matrix, which are thermal sensitive; hence, higher temperatures and extraction times may cause their degradation and, consequently, decrease the antioxidant capacity [17]. The optimal percentage of ethanol proposed by the Multiple Response Optimization was similar to that obtained by Karami et al. for the MAE extraction technique to obtain phenolic compounds from licorice root, which is rich in flavonoids. This study revealed that this percentage of ethanol-water gave an optimal extraction of flavonoids due to the physicochemical characteristics of these solvents [36].

Considering the optimal extraction conditions for Multiple Response Optimization for *L. citriodora*, the necessary temperature is very low, around 20 °C, extraction time around 7 min and a very high percentage of ethanol, 95%. This very low temperature may be due to the fact that as the PLE extraction technique uses high pressures, the high pressure enables the solvent penetration in the sample, allowing extraction at lower temperatures and maintaining the stability of the most thermosensitive compounds [43]. Regarding the optimal percentage of ethanol, compounds with high antioxidant capacity, such as verbascoside and its isomers, were extracted with higher ethanol concentrations [44].

## 4. Conclusions

The extraction conditions using the MAE technique for *H. sabdariffa* calyces and PLE technique for *L. citriodora* leaves were optimized to maximize the response variables total phenolic compounds by HPLC-ESI-TOF/MS, TPC by the Folin-Ciocalteu test, and two variables that measure antioxidant capacity, FRAP and TEAC. The quadratic model applied in the *H. sabdariffa* design represented a very good approximation in the variables of the Folin-Ciocalteu test and in the two antioxidant capacity variables. However, for this matrix, Total Polar Compounds presented a slight deviation to the proposed model. In contrast, the quadratic model proposed in the design for *L. citriodora* represented a very good approximation for the four response variables evaluated. Through the statistical analysis, it was possible to know the influence of temperature, ethanol percentage and time to allow optimal conditions, maximizing the dependent variables studied. In order to obtain reliable results in the multiple response analysis performed in *H. sabdariffa,* it was deemed appropriate to eliminate the response variable that the model had not adequately adjusted. The results demonstrated that moderate temperature and high ethanol percentage induced the recovery of greater concentration of flavonoids when MAE was applied, achieving better antioxidant results. In the case of *L. citriodora,* the multiple response analysis included the four study variables. The results revealed that the low temperature and a high percentage of ethanol were needed to obtain high concentration of antioxidant compounds. Considering the evaluated results, it is possible to affirm the importance of multiple response analyses to develop nutraceuticals or functional foods, since it has to be approached from a wide point of view; not only the total content of phenolic compounds or their bioactivity, but also both variables should be evaluated to optimize its production. Furthermore, as confirmed by this study, these analyses can be extrapolated in different techniques and in different matrices with phenolic compounds from different families.

## Figures and Tables

**Table 1:** Results of the response variables Total Polar Compounds, TPC, FRAP and TEAC for each condition in *H. sabdariffa* MAE extracts.

Experimental Design Condition	Total Polar Compounds ^a,1^	TPC ^b^	FRAP ^c^	TEAC ^d^
1	92.514 ± 4.727	41.114 ± 1.832	0.935 ± 0.036	0.254 ± 0.009
2	76.171 ± 1.501	57.953 ± 2.591	0.959 ± 0.044	0.260 ± 0.002
3	56.469 ± 3.547	39.822 ± 0.006	0.767 ± 0.024	0.213 ± 0.004
4	96.682 ± 1.676	72.634 ± 5.393	0.781 ± 0.062	0.268 ± 0.009
5	83.807 ± 1.365	52.772 ± 4.487	0.995 ± 0.034	0.284 ± 0.008
6	84.987 ± 1.934	43.273 ± 3.957	0.757 ± 0.043	0.216 ± 0.004
7	58.774 ± 1.767	25.570 ± 1.832	0.651 ± 0.027	0.182 ± 0.005
8	87.112 ± 4.804	78.679 ± 2.591	0.711 ± 0.054	0.228 ± 0.013
9	81.339 ± 1.511	45.864 ± 1.496	0.799 ± 0.016	0.233 ± 0.003
10	50.027 ± 1.107	26.002 ± 1.496	0.524 ± 0.011	0.133 ± 0.006
11	201.466 ± 5.356	61.408 ± 2.991	0.819 ± 0.024	0.285 ± 0.010
12	53.368 ± 1.331	40.682 ± 1.496	0.537 ± 0.024	0.166 ± 0.003
13	56.314 ± 0.951	44.136 ± 3.957	0.607 ± 0.024	0.190 ± 0.001
14	77.399 ± 0.682	74.361 ± 5.983	0.968 ± 0.022	0.280 ± 0.006
15	71.110 ± 0.937	54.499 ± 2.991	0.995 ± 0.018	0.261 ± 0.001
16	69.784 ± 1.262	42.407 ± 0.003	0.903 ± 0.043	0.232 ± 0.003

^a^ Expressed in mg total polar compounds/g of dry extract; ^b^ Expressed in mg Gallic acid equivalents/g of dry extract; ^c^ Expressed in mmol FeSO_4_ equivalents/g of dry extract; ^d^ Expressed in mmol Trolox equivalents/g of dry extract; ^1^ Adapted from Pimentel Moral et al. [17]; TPC: Folin-Ciocalteu; FRAP: Ferric Reducing Antioxidant Power; TEAC: Trolox Equivalent Antioxidant Capacity.

**Table 2:** Major compounds tentatively identified in *H. sabdariffa* calyces by high-performance liquid chromatography coupled to mass spectrometry (HPLC-ESI-TOF-MS).

Molecular Formula	*m/z* Calculated	Compound
C_6_H_8_O_8_	207.0146	Hydroxicitric acid
C_6_H_6_O_7_	189.0041	Hibiscus acid
C_8_H_12_O_8_	235.0459	Hibiscus acid hydroxyethylester
C_8_H_10_O_7_	217.0354	Hibiscus acid dimethylester
C_16_H_18_O_9_	353.0878	Neochlorogenic acid
C_16_H_18_O_9_	353.0878	Chlorogenic acid
C_16_H_18_O_9_	353.0878	Cryptochlorogenic acid
C_15_H_12_O_9_	335.0409	Methyl digallate
C_16_H_18_O_8_	337.0929	Coumaroilquinic acid
C_16_H_20_O_10_	371.0984	Dihydroferulic acid-4-*O*-glucuronide
C_26_H_28_O_16_	595.1305	Quercetin-3-sambubioside
C_17_H_30_O_16_	489.1461	Quercetin-3-rutinoside
C_16_H_16_O_8_	335.0772	5-*O*-caffeoylshikimic acid
C_21_H_20_O_12_	463.0882	Quercetin-3-glucoside
C_18_H_22_O_9_	381.1191	Ethylchlorogenate
C_18_H_22_O_9_	381.1191	Ethylchlorogenate isomer II
C_16_H_16_O_7_	319.0823	Methylepigallocatechin
C_15_H_10_O_8_	317.0303	Myricetin
C_18_H_19_NO_4_	312.1241	*N*-feruloyltiramine
C_15_H_10_O_7_	301.0354	Quercetin
C_15_ H_10_O_6_	285.0405	Kaempferol

**Table 3:** Results of the response variables for each condition in *L. citriodora*. Response variables: Total Polar Compounds, TPC, FRAP and TEAC.

Experimental Design Condition	Total Polar Compounds ^a,1^	TPC ^b^	FRAP ^c^	TEAC ^d^
1	304.734 ± 21.965	181.442 ± 9.098	1.720 ± 0.080	0.64 ± 0.028
2	369.613 ± 24.362	253.117 ± 1.496	2.402 ± 0.131	0.553 ± 0.054
3	213.096 ± 13.480	168.489 ± 5.393	2.095 ± 0.048	0.580 ± 0.068
4	220.119 ± 11.880	195.259 ± 4.487	1.997 ± 0.063	0.524 ± 0.005
5	199.962 ± 9.621	159.853 ± 10.786	1.745 ± 0.098	0.653 ± 0.053
6	268.933 ± 17.803	235.846 ± 7.915	1.802 ± 0.058	0.553 ± 0.019
7	228.959 ± 17.110	197.850 ± 2.591	1.875 ± 0.120	0.438 ± 0.022
8	221.533 ± 12.409	227.211 ± 12.779	2.313 ± 0.055	0.574 ± 0.029
9	141.768 ± 9.291	145.173 ± 7.479	1.685 ± 0.153	0.555 ± 0.049
10	252.724 ± 10.062	219.439 ± 3.957	1.615 ± 0.125	0.553 ± 0.031
11	329.635 ± 20.249	193.532 ± 3.957	2.252 ± 0.085	0.532 ± 0.085
12	226.461 ± 8.706	189.214 ± 1.496	1.728 ± 0.111	0.420 ± 0.006
13	155.232 ± 28.764	211.667 ± 1.496	1.877 ± 0.069	0.493 ± 0.024
14	282.828 ± 16.771	242.755 ± 6.520	2.318 ± 0.031	0.665 ± 0.002
15	125.646 ± 5.013	185.760 ± 7.915	1.230 ± 0.048	0.453 ± 0.022
16	96.830 ± 4.096	163.307 ± 1.496	1.590 ± 0.056	0.558 ± 0.088

^a^ Expressed in mg total polar compounds/g of dry extract; ^b^ Expressed in mg Gallic acid equivalents/g of dry extract; ^c^ Expressed in mmol FeSO_4_ equivalents/g of dry extract; ^d^ Expressed in mmol Trolox equivalents/g of dry extract; ^1^ Adapted from Leyva-Jiménez et al. [14] with some modifications; TPC: Folin-Ciocalteu; FRAP: Ferric Reducing Antioxidant Power; TEAC: Trolox Equivalent Antioxidant Capacity.

**Table 4:** Major compounds tentatively identified in *L. citriodora* leaves by high-performance liquid chromatography coupled to mass spectrometry (HPLC-ESI-TOF-MS).

Molecular Formula	*m/z* Calculated	Compound
C_6_H_11_O_7_	195.0510	Gluconic acid
C_16_H_21_O_10_	373.1140	Gardoside
C_16_H_19_O_11_	387.0933	Ixoside
C_20_H_29_O_12_	461.1664	Verbasoside
C_21_H_27_O_13_	487.1457	Cistanoside F
C_16_H_21_O_11_	389.1089	Theveside
C_18_H_27_O_9_	387.1661	Tuberonic acid glucoside
C_29_H_37_O_16_	641.2087	β Hydroxyverbascoside derivative
C_29_H_37_O_16_	641.2087	β Hydroxyisoverbascoside derivative
C_29_H_35_O_16_	639.1931	β Hydroxyverbascoside
C_27_H_25_O_18_	637.1140	Luteolin-7-diglucoronide
C_29_H_35_O_16_	639.1931	β Hydroxyisoverbascoside
C_27_H_25_O_17_	621.1097	Apigenin-7-diglucoronide
C_25_H_29_O_13_	537.1614	Lippioside I
C_30_H_37_O_16_	653.2087	Campneoside I
C_28_H_27_O_18_	651.1355	Chrysoeriol-7-diglucuronide
C_29_H_35_O_15_	623.1981	Verbascoside
C_26_H_33_O_11_	521.2028	Lariciresinol glucopyranoside
C_29_H_35_O_15_	623.1981	Isoverbascoside
C_29_H_35_O_15_	623.1981	Forsythoside A
C_25_H_29_O_12_	521.1664	Hydroxycampsiside
C_26_H_31_O_13_	551.1770	Durantoside I
C_30_H_37_O_15_	637.2138	Leucoseptoside A
C_28_H_27_O_17_	635.1254	Acacetin-7-diglucoronide
C_31_H_39_O_15_	651.2294	Martynoside
C_29_H_35_O_13_	591.2083	Osmanthuside B
C_16_H_11_O_6_	299.0561	Dimethyl kaempferol
C_17_H_13_O_7_	329.0667	Dimethyl quercetin

**Table 5:** Analysis of variance (ANOVA) of the regression models in *H. sabdariffa*. Response variables: Total Polar Compounds, TPC, FRAP and TEAC.

Source	Total Polar Compounds	TPC	FRAP	TEAC
	*p* Value	*p* Value	*p* Value	*p* Value
X_1_: Temperature	0.1796	0.0306 ^a^	0.0798 ^b^	0.6682
X_2_: Solvent	0.2141	0.0003 ^a^	0.0131 ^a^	0.0118 ^a^
X_3_: Time	0.7662	0.0459 ^a^	0.8848	0.9187
X_1_ X_1_	0.2567	0.6735	0.2945	0.6745
X_1_ X_2_	0.8091	0.0078 ^a^	0.7945	0.4297
X_1_ X_3_	0.9191	0.9815	0.8184	0.9240
X_2_ X_2_	0.1766	0.9960	0.1263	0.1806
X_2_ X_3_	0.7743	0.8339	0.7707	0.6862
X_3_ X_3_	0.2769	0.9588	0.5047	0.4107
Model value	0.2631	0.0004 ^a^	0.0101 ^a^	0.0168 ^a^
*R* ^2^	0.621	0.937	0.784	0.740
CV	<29.64	<12.79	<13.81	<13.50

*R*^2^ = Quadratic correlation coefficient; CV = Coefficient of variation (%); ^a^ Significant (*p* < 0.050); ^b^ Marginally significant (*p* < 0.100); TPC: Folin-Ciocalteu; FRAP: Ferric Reducing Antioxidant Power; TEAC: Trolox Equivalent Antioxidant Capacity.

**Table 6:** Values of the independent conditions to optimize the value of the response variables in *H. sabdariffa*. Response variables: Total Polar Compounds, TPC, FRAP and TEAC.

Source	Total Polar Compounds	TPC	FRAP	TEAC
X_1_: Temperature ^a^	164.36	164.36	64.91	118.15
X_2_: Solvent ^b^	57.33	83.62	67.08	72.56
X_3_: Time ^c^	13.15	2.85	11.70	13.43
Theoretical optimum value	150.456 ^d^	97.742 ^e^	0.991 ^f^	0.278 ^g^

^a^ Expressed in °C; ^b^ Expressed in % of ethanol; ^c^ Expressed in minutes; ^d^ Expressed in mg total polar compounds/g of dry extract; ^e^ Expressed in mg Gallic acid equivalents/g of dry extract; ^f^ Expressed in mmol FeSO_4_ equivalents/g of dry extract; ^g^ Expressed in mmol Trolox equivalents/g of dry extract; TPC: Folin-Ciocalteu; FRAP: Ferric Reducing Antioxidant Power; TEAC: Trolox Equivalent Antioxidant Capacity.

**Table 7:** Analysis of variance (ANOVA) of the regression models in *L. citriodora*. Response variables: Total Polar Compounds, TPC, FRAP and TEAC.

Source	Total Polar Compounds	TPC	FRAP	TEAC
	*p* Value	*p* Value	*p* Value	*p* Value
X_1_: Temperature	0.0001 ^a^	0.0241 ^a^	0.0412 ^a^	0.0511 ^b^
X_2_: Solvent	0.0023 ^a^	0.0009 ^a^	0.0556 ^b^	0.1760
X_3_: Time	0.4840	0.9881	0.9649	0.4579
X_1_ X_1_	0.1580	0.2192	0.0424 ^a^	0.4636
X_1_ X_2_	0.4944	0.7579	0.2290	0.9206
X_1_ X_3_	0.0430 ^a^	0.0980 ^b^	0.7339	0.5773
X_2_ X_2_	0.2570	0.1079	0.1320	0.0869 ^b^
X_2_ X_3_	0.0520 ^b^	0.1040	0.8596	0.7657
X_3_ X_3_	0.9450	0.0315 ^a^	0.9599	0.2396
Model value	0.0001 ^a^	0.0057 ^a^	0.0852 ^b^	0.0892 ^b^
*R* ^2^	0.955	0.918	0.800	0.725
CV	<15.12	<7.22	<13.04	<10.09

*R*^2^ = Quadratic correlation coefficient; CV = Coefficient of variation (%); ^a^ Significant (*p* < 0.050); ^b^ Marginally significant (*p* < 0.100); TPC: Folin-Ciocalteu; FRAP: Ferric Reducing Antioxidant Power; TEAC: Trolox Equivalent Antioxidant Capacity.

**Table 8:** Values of the independent conditions to optimize the value of the response variables in *L. citriodora*. Response variables: Total Polar Compounds, TPC, FRAP and TEAC.

Source	Total Polar Compounds	TPC	FRAP	TEAC
X_1_: Temperature ^a^	26.51	19.90	71.12	26.25
X_2_: Solvent ^b^	95.05	95.05	95.05	4.95
X_3_: Time ^c^	2.85	6.93	22.05	11.47
Theoretical optimum value	374.344 ^d^	252.952 ^e^	2.541 ^f^	0.696 ^g^

^a^ Expressed in °C; ^b^ Expressed in % of ethanol; ^c^ Expressed in minutes; ^d^ Expressed in mg total polar compounds/g of dry extract; ^e^ Expressed in mg Gallic acid equivalents/g of dry extract; ^f^ Expressed in mmol FeSO_4_ equivalents/g of dry extract; ^g^ Expressed in mmol Trolox equivalents/g of dry extract; TPC: Folin-Ciocalteu; FRAP: Ferric Reducing Antioxidant Power; TEAC: Trolox Equivalent Antioxidant Capacity.

**Table 9:** Multiple response optimization analysis in *H. sabdariffa* and *L. citriodora*.

***H. sabdariffa* Multiple Response Optimization with Total Polar Compounds, TPC, FRAP and TEAC (Optimum Desirability = 1.0)**					
**Independent variables**		**TPC**	**FRAP**		**TEAC**
Temperature ^a^	122.25	78.963 ^d^	0.870 ^e^		0.249 ^f^
Solvent ^b^	78.58				
Time ^c^	2.98				
***L. citriodora* Multiple Response Optimization with TPC, FRAP and TEAC (Optimum Desirability = 0.99)**					
**Independent variables**		**Total Polar Compounds**	**TPC**	**FRAP**	**TEAC**
Temperature ^a^	19.90	373.449 ^g^	252.953 ^d^	2.432 ^e^	0.595 ^f^
Solvent ^b^	95.05				
Time ^c^	6.94				

^a^ Expressed in °C; ^b^ Expressed in % of ethanol; ^c^ Expressed in minutes; ^d^ Expressed in mg Gallic acid equivalents/g of dry extract; ^e^ Expressed in mmol FeSO_4_ equivalents/g of dry extract; ^f^ Expressed in mmol Trolox equivalents/g of dry extract; ^g^ Expressed in mg total polar compounds/g of dry extract; TPC: Folin-Ciocalteu; FRAP: Ferric Reducing Antioxidant Power; TEAC: Trolox Equivalent Antioxidant Capacity.

## Supplementary Materials

The following are available online at https://www.mdpi.com/2076-3921/9/12/1175/s1, Table S1: MAE factorial design 2^3^ experimental values of tested independent variables, Table S2: PLE factorial design 2^3^ experimental values of tested independent variables, Equation (S1): Regression model equations of *H. sabdariffa*, Equation (S2): Regression model equations of *L. citriodora*.

## References

[1] Gul K., Singh A.K., Jabeen R. (2016). Nutraceuticals and Functional Foods: The Foods for the Future World. Crit. Rev. Food Sci. Nutr..

[2] Santos-Lozano J.M., Rada M., Lapetra J., Guinda Á., Jiménez-Rodríguez M.C., Cayuela J.A., Ángel-Lugo A., Vilches-Arenas Á., Gómez-Martín A.M., Ortega-Calvo M. (2019). Prevention of type 2 diabetes in prediabetic patients by using functional olive oil enriched in oleanolic acid: The PREDIABOLE study, a randomized controlled trial. Diabetes Obes. Metab..

[3] Katsube T., Yamasaki M., Shiwaku K., Ishijima T., Matsumoto I., Abe K., Yamasaki Y. (2010). Effect of flavonol glycoside in mulberry (*Morus alba* L.) leaf on glucose metabolism and oxidative stress in liver in diet-induced obese mice. J. Sci. Food Agric..

[4] Sedlak L., Wojnar W., Zych M., Wyględowska-Promieńska D., Mrukwa-Kominek E., Kaczmarczyk-Sedlak I. (2018). Effect of Resveratrol, a Dietary-Derived Polyphenol, on the Oxidative Stress and Polyol Pathway in the Lens of Rats with Streptozotocin-Induced Diabetes. Nutrients.

[5] de la Luz Cádiz-Gurrea M., Olivares-Vicente M., Herranz-López M., Román-Arráez D., Fernández-Arroyo S., Micol V., Segura-Carretero A. (2018). Bioassay-guided purification of Lippia citriodora polyphenols with AMPK modulatory activity. J. Funct. Foods.

[6] Mezni A., Mhadhbi L., Khazri A., Sellami B., Dellali M., Mahmoudi E., Beyrem H. (2020). The protective effect of *Hibiscus sabdariffa* calyxes extract against cypermethrin induced oxidative stress in mice. Pestic. Biochem. Physiol..

[7] Herranz-López M., Olivares-Vicente M., Encinar J.A., Barrajón-Catalán E., Segura-Carretero A., Joven J., Micol V. (2017). Multi-targeted molecular effects of *Hibiscus sabdariffa* polyphenols: An opportunity for a global approach to obesity. Nutrients.

[8] Mojiminiyi F.B.O., Dikko M., Muhammad B.Y., Ojobor P.D., Ajagbonna O.P., Okolo R.U., Igbokwe U.V., Mojiminiyi U.E., Fagbemi M.A., Bello S.O. (2007). Antihypertensive effect of an aqueous extract of the calyx of *Hibiscus sabdariffa*. Fitoterapia.

[9] Ali R.F.M., El-Anany A.M. (2017). Hypolipidemic and hypocholesterolemic effect of roselle (*Hibiscus sabdariffa* L.) seeds oil in experimental male rats. J. Oleo Sci..

[10] Villegas-Aguilar M.D.C., Fernández-Ochoa Á., Cádiz-Gurrea M.D.L.L., Pimentel-Moral S., Lozano-Sánchez J., Arráez-Román D., Segura-Carretero A. (2020). Pleiotropic biological effects of dietary phenolic compounds and their metabolites on energy metabolism, inflammation and aging. Molecules.

[11] Ezzat S.M., Salama M.M., Seif el-Din S.H., Saleh S., El-Lakkany N.M., Hammam O.A., Salem M.B., Botros S.S. (2016). Metabolic profile and hepatoprotective activity of the anthocyanin-rich extract of *Hibiscus sabdariffa* calyces. Pharm. Biol..

[12] Leyva-Jiménez F.J., Lozano-Sánchez J., de la Luz Cádiz-Gurrea M., Arráez-Román D., Segura-Carretero A. (2019). Functional ingredients based on nutritional phenolics. A case study against inflammation: *Lippia* genus. Nutrients.

[13] Rúa J., López-Rodríguez I., Sanz J., del Valle Fernández P., Garcia M.D.C., Garcia Armesto M.R. (2019). Antimicrobial efficacy of *Lippia citriodora* natural extract against *Escherichia coli* and *Enterococcus faecalis* in “Piel de Sapo” melon juice. Food Sci. Nutr..

[14] Leyva-Jiménez F.J., Lozano-Sánchez J., Borrás-Linares I., Arráez-Román D., Segura-Carretero A. (2018). Comparative study of conventional and pressurized liquid extraction for recovering bioactive compounds from *Lippia citriodora* leaves. Food Res. Int..

[15] Cádiz-Gurrea M., Lozano-Sánchez J., Fernández-Ochoa Á., Segura-Carretero A. (2019). Enhancing the Yield of Bioactive Compounds from *Sclerocarya birrea* Bark by Green Extraction Approaches. Molecules.

[16] Yu W., Chen H., Xiang Z., He N. (2019). Preparation of Polysaccharides from *Ramulus mori*, and Their Antioxidant, Anti-Inflammatory and Antibacterial Activities. Molecules.

[17] Pimentel-Moral S., Borrás-Linares I., Lozano-Sánchez J., Arráez-Román D., Martínez-Férez A., Segura-Carretero A. (2018). Microwave-assisted extraction for *Hibiscus sabdariffa* bioactive compounds. J. Pharm. Biomed. Anal..

[18] De Biaggi M., Donno D., Mellano M.G., Riondato I., Rakotoniaina E.N., Beccaro G.L. (2018). *Cornus mas* (L.) Fruit as a Potential Source of Natural Health-Promoting Compounds: Physico-Chemical Characterisation of Bioactive Components. Plant Foods Hum. Nutr..

[19] Pimentel-Moral S., Borrás-Linares I., Lozano-Sánchez J., Arráez-Román D., Martínez-Férez A., Segura-Carretero A. (2018). Supercritical CO_2_ extraction of bioactive compounds from *Hibiscus sabdariffa*. J. Supercrit. Fluids.

[20] Hu M., Yan H., Fu Y., Jiang Y., Yao W., Yu S., Zhang L., Wu Q., Ding A., Shan M. (2019). Optimal extraction study of gastrodin-type components from gastrodia elata tubers by response surface design with integrated phytochemical and bioactivity evaluation. Molecules.

[21] Chen H., Xiao H., Pang J. (2020). Parameter optimization and potential bioactivity evaluation of a betulin extract from white birch bark. Plants.

[22] Cádiz-Gurrea M.D.L.L., Borrás-Linares I., Lozano-Sánchez J., Joven J., Fernández-Arroyo S., Segura-Carretero A. (2017). Cocoa and grape seed byproducts as a source of antioxidant and anti-inflammatory proanthocyanidins. Int. J. Mol. Sci..

[23] Benzie I.F.F., Strain J.J. (1996). The Ferric Reducing Ability of Plasma (FRAP) as a Measure of “Antioxidant Power”: The FRAP Assay. Anal. Biochem..

[24] Miller N.J., Rice-Evans C., Davies M.J., Gopinathan V., Milner A. (1993). A novel method for measuring antioxidant capacity and its application to monitoring the antioxidant status in premature neonates. Clin. Sci..

[25] de la Luz Cádiz-Gurrea M., Fernández-Arroyo S., Joven J., Segura-Carretero A. (2013). Comprehensive characterization by UHPLC-ESI-Q-TOF-MS from an *Eryngium bourgatii* extract and their antioxidant and anti-inflammatory activities. Food Res. Int..

[26] Skroza D., Generalić Mekinić I., Svilović S., Šimat V., Katalinić V. (2015). Investigation of the potential synergistic effect of resveratrol with other phenolic compounds: A case of binary phenolic mixtures. J. Food Compos. Anal..

[27] Sánchez-Marzo N., Lozano-Sánchez J., Cádiz-Gurrea M.D.L.L., Herranz-López M., Micol V., Segura-Carretero A. (2019). Relationships between chemical structure and antioxidant activity of isolated phytocompounds from lemon verbena. Antioxidants.

[28] Everette J.D., Bryant Q.M., Green A.M., Abbey Y.A., Wangila G.W., Walker R.B. (2010). Thorough study of reactivity of various compound classes toward the folin-Ciocalteu reagent. J. Agric. Food Chem..

[29] Nobossé P., Fombang E.N., Mbofung C.M.F. (2018). Effects of age and extraction solvent on phytochemical content and antioxidant activity of fresh *Moringa oleifera* L. leaves. Food Sci. Nutr..

[30] Xiao X., Song W., Wang J., Li G. (2012). Microwave-assisted extraction performed in low temperature and in vacuo for the extraction of labile compounds in food samples. Anal. Chim. Acta.

[31] Firuzi O., Lacanna A., Petrucci R., Marrosu G., Saso L. (2005). Evaluation of the antioxidant activity of flavonoids by “ferric reducing antioxidant power” assay and cyclic voltammetry. Biochim. Biophys. Acta-Gen. Subj..

[32] Orak H.H., Magdalena K., Amarowicz R., Orak A., Penkacik K. (2019). Genotype-Related Differences in the Phenolic Compound Profile and Antioxidant Activity of Extracts from Olive (*Olea europaea* L.) Leaves. Molecules.

[33] Sanhueza L., Melo R., Montero R., Maisey K., Mendoza L., Wilkens M. (2017). Synergistic interactions between phenolic compounds identified in grape pomace extract with antibiotics of different classes against *Staphylococcus aureus* and *Escherichia coli*. PLoS ONE.

[34] Gutiérrez Pulido H., de la Vara Salazar R., Intergovernmental Panel on Climate Change (2004). Análisis y Diseño de Experimentos.

[35] Liyana-Pathirana C., Shahidi F. (2005). Optimization of extraction of phenolic compounds from wheat using response surface methodology. Food Chem..

[36] Karami Z., Emam-Djomeh Z., Mirzaee H.A., Khomeiri M., Mahoonak A.S., Aydani E. (2015). Optimization of microwave assisted extraction (MAE) and soxhlet extraction of phenolic compound from licorice root. J. Food Sci. Technol..

[37] Mendes M., Carvalho A.P., Magalhães J.M.C.S., Moreira M., Guido L., Gomes A.M., Delerue-Matos C. (2016). Response surface evaluation of microwave-assisted extraction conditions for *Lycium barbarum* bioactive compounds. Innov. Food Sci. Emerg. Technol..

[38] Bahorun T., Luximon-Ramma A., Crozier A., Aruoma O.I. (2004). Total phenol, flavonoid, proanthocyanidin and vitamin C levels and antioxidant activities of Mauritian vegetables. J. Sci. Food Agric..

[39] Csepregi K., Neugart S., Schreiner M., Hideg É. (2016). Comparative evaluation of total antioxidant capacities of plant polyphenols. Molecules.

[40] DeGraft-Johnson J., Kolodziejczyk K., Krol M., Nowak P., Krol B., Nowak D. (2007). Ferric-reducing ability power of selected plant polyphenols and their metabolites: Implications for clinical studies on the antioxidant effects of fruits and vegetable consumption. Basic Clin. Pharmacol. Toxicol..

[41] Cesari L., Namysl S., Canabady-Rochelle L., Mutelet F. (2017). Phase equilibria of phenolic compounds in water or ethanol. Fluid Phase Equilib..

[42] Yolmeh M., Jafari S.M. (2017). Applications of Response Surface Methodology in the Food Industry Processes. Food Bioprocess Technol..

[43] Tripodo G., Ibáñez E., Cifuentes A., Gilbert-López B., Fanali C. (2018). Optimization of pressurized liquid extraction by response surface methodology of Goji berry (*Lycium barbarum* L.) phenolic bioactive compounds. Electrophoresis.

[44] Rosa A.D., Junges A., Fernandes I.A., Cansian R.L., Corazza M.L., Franceschi E., Backes G.T., Valduga E. (2019). High pressure extraction of olive leaves (*Olea europaea*): Bioactive compounds, bioactivity and kinetic modelling. J. Food Sci. Technol..

