# Supplementary material for: Comprehensive Analysis of Antioxidant Compounds from Lippia citriodora and Hibiscus sabdariffa Green Extracts Attained by Response Surface Methodology
Source: Antioxidants (Basel). 2020 Nov 25;9(12):1175. doi: 10.3390/antiox9121175 (PMC7761067; doi:10.3390/antiox9121175)
Supplement: Supplementary file 1 [file antioxidants-09-01175-s001.pdf]

antioxidants

Article

Comprehensive analysis of antioxidant compounds from *Lippia citriodora* and *Hibiscus sabdariffa* Green extracts attained by Response Surface Methodology.

María del Carmen Villegas-Aguilar [1,2], Francisco Javier Leyva-Jiménez [2], María de la Luz Cádiz-Gurrea [1,2,*], Antonio Segura-Carretero [1,2,†] and David Arráez-Román [1,2,*,†]

[1] Department of Analytical Chemistry, University of Granada, Spain; marivillegas@ugr.es (M.d.C.V.-A.); ansegura@ugr.es (A.S.-C.)
[2] Research and Development of Functional Food Centre (CIDAF), Granada, Spain; jleyva@cidaf.es (F.J.L.-J.)
* Correspondence: mluzcadiz@ugr.es (M.d.l.L.C.-G.); darraez@ugr.es (D.A.-R.)
† These authors shared author co-seniorship.

Received: date; Accepted: date; Published: date

Supplementary

Table S1. MAE factorial design 2^3 experimental values of tested independent variables.

RUN	TEMPERATURE (°C)	TIME (MIN)	SOLVENT (% ETHANOL)
**1**	50	20	75
**2**	50	5	75
**3**	50	20	15
**4**	150	20	75
**5**	100	12.5	45
**6**	100	12.5	45
**7**	100	12.5	6
**8**	150	5	75
**9**	100	22	45
**10**	150	20	15
**11**	164	12.5	45
**12**	150	5	15
**13**	50	5	15
**14**	100	12.5	84
**15**	100	3	45
**16**	36	12.5	45

Table S2. PLE factorial design 2^3 experimental values of tested independent variables.

RUN	TEMPERATURE (°C)	TIME (MIN)	SOLVENT (% ETHANOL)
**1**	40	20	15
**2**	40	5	85
**3**	110	12.5	5
**4**	110	22	50
**5**	40	5	15
**6**	20	12.5	50
**7**	110	3	50
**8**	110	12.5	50
**9**	180	5	15
**10**	110	12.5	50
**11**	40	20	85
**12**	180	5	85
**13**	180	20	85
**14**	110	12.5	95
**15**	200	12.5	50
**16**	180	20	15

Equation S1. Regression model equations of *H. sabdariffa.*

$$\text{Total Polar Compounds} = 27.156 - 1.207X_1 + 2.308X_2 + 7.817X_3 + 0.007X_1X_1 + 0.002X_1X_2 - 0.003X_1X_3 - 0.025X_2X_2 + 0.016X_2X_3 - 0.311X_3X_3$$

$$\text{Folin-Ciocalteu} = 55.084 - 0.252X_1 - 0.075X_2 - 0.605X_3 + 0.0004X_1X_1 + 0.006X_1X_2 + 0.0001X_1X_3 + 0.00002X_2X_2 - 0.002X_2X_3 + 0.003X_3X_3$$

$$\text{FRAP} = 0.275 + 0.004X_1 + 0.014X_2 + 0.020X_3 - 0.00002X_1X_1 - 0.000007X_1X_2 - 0.00003X_1X_3 - 0.0001X_2X_2 - 0.00006X_2X_3 - 0.0006X_3X_3$$

$$\text{TEAC} = 0.139 + 0.0002X_1 + 0.003X_2 + 0.005X_3 - 0.000003X_1X_1 + 0.000007X_1X_2 - 0.000003X_1X_3 - 0.00003X_2X_2 + 0.00002X_2X_3 - 0.0002X_3X_3$$

Equation S2. Regression model equations of *L. citriodora*.

Total Polar Compounds = $150.657 + 0.473X_1 + 1.295X_2 + 8.413X_3 – 0.004X_1X_1 – 0.003X_1X_2 – 0.043X_1X_3 + 0.011X_2X_2 – 0.081X_2X_3 – 0.014X_3X_3$

Folin-Ciocalteu = $115.366 – 0.008X_1 + 2.205X_2 + 7.278X_3 – 0.002X_1X_1 – 0.001X_1X_2 + 0.019X_1X_3 – 0.009X_2X_2 – 0.037X_2X_3 – 0.301X_3X_3$

FRAP = $1.598 + 0.010X_1 – 0.005X_2 – 0.011X_3 – 0.00005X_1X_1 – 0.00004X_1X_2 + 0.00005X_1X_3 + 0.0001X_2X_2 + 0.00006X_2X_3 + 0.00009X_3X_3$

TEAC = $0.649 – 0.00006X_1 – 0.005X_2 + 0.013X_3 – 0.000004X_1X_1 + 8{,}962E^{-7}X_1X_2 + 0.00002X_1X_3 + 0.00004X_2X_2 + 0.00003X_2X_3 - 0.0006X_3X_3$

© 2020 by the authors. Submitted for possible open access publication under the terms and conditions of the Creative Commons Attribution (CC BY) license (http://creativecommons.org/licenses/by/4.0/).
